# Benzimidazole Containing Acetamide Derivatives Attenuate Neuroinflammation and Oxidative Stress in Ethanol-Induced Neurodegeneration

**DOI:** 10.3390/biom10010108

**Published:** 2020-01-08

**Authors:** Muhammad Imran, Lina Tariq Al Kury, Humaira Nadeem, Fawad Ali Shah, Muzaffar Abbas, Shagufta Naz, Arif-ullah Khan, Shupeng Li

**Affiliations:** 1 Department of Pharmaceutical Chemistry, Faculty of Pharmaceutical Sciences, Riphah International University, Islamabad 44000, Pakistan; m.imran.khanzada@gmail.com (M.I.); shagufta.naz@riphah.edu.pk (S.N.); 2 College of Natural and Health Sciences, Zayed University Abu Dhabi, Abu Dhabi 144534, UAE; Lina.AlKury@zu.ac.ae; 3 Department of Pharmacology, Faculty of Pharmaceutical Sciences, Riphah International University, Islamabad 747424, Pakistan; arif.ullah@riphah.edu.pk; 4 Department of Pharmacy, Capital University of Science & Technology, Islamabad Expressway, Islamabad 747424, Pakistan; mabbas14@gmail.com; 5 State Key Laboratory of Oncogenomics, School of Chemical Biology and Biotechnology, Peking University Shenzhen Graduate School, Shenzhen 518055, China; lisp@pku.edu.cn

**Keywords:** oxidative stress, neuroinflammation, docking, neuroprotective, cortex, hippocampus, ethanol

## Abstract

Oxidative stress-induced neuroinflammation is the prominent feature of neurodegenerative disorders, and is characterized by a gradual decline of structure and function of neurons. Many biochemical events emerge thanks to the result of this neurodegeneration, and ultimately provoke neuroinflammation, activation of microglia, and oxidative stress, leading to neuronal death. This cascade not only explains the complexity of events taking place across different stages, but also depicts the need for more effective therapeutic agents. The present study was designed to investigate the neuroprotective effects of newly synthesized benzimidazole containing acetamide derivatives, 3a (2-(4-methoxyanilino)-N-[1-(4-methylbenzene-1-sulfonyl)-1H-benzimidazol-2-yl] acetamide) and 3b (2-(Dodecylamino)-N-[1-(4-methylbenzene-1-sulfonyl)-1H-benzimidazol-2-yl] acetamide) against ethanol-induced neurodegeneration in the rat model. Both derivatives were characterized spectroscopically by proton NMR (^1^H-NMR) and carbon-13 NMR (^13^C-NMR) and evaluated for neuroprotective potential using different pharmacological approaches. In vivo experiments demonstrated that ethanol triggered neurodegeneration characterized by impaired antioxidant enzymes and elevated oxidative stress. Furthermore, ethanol administration induced neuroinflammation, as demonstrated by elevated expression of tumor necrotic factor (TNF-α), nuclear factor κB (NF-κB), cyclooxygenase-2 (COX2), and ionized calcium-binding adapter molecule-1 (Iba-1), which was further validated by enzyme-linked immunosorbent assay (ELISA). Treatment with 3a and 3b ameliorated the ethanol-induced oxidative stress, neuroinflammation, and memory impairment. The affinity of synthesized derivatives towards various receptors involved in neurodegeneration was assessed through docking analysis. The versatile nature of benzimidazole nucleus and its affinity toward several receptors suggested that it could be a multistep targeting neuroprotectant. As repetitive clinical trials of neuroprotectants targeting a single step of the pathological process have failed previously, our results suggested that a neuroprotective strategy of acting at different stages may be more advantageous to intervene in the vicious cycles of neuroinflammation.

## 1. Introduction

Neurodegeneration is the gradual decline of the structure and function of neurons. Inflammation and oxidative stress are pivotal mediators in the development of these neurodegenerative diseases such as Alzheimer’s disease (AD), Parkinson’s disease, and ischemic brain injury [1,2,3]. Many other biochemical events such as loss of ionic gradient, the release of excitatory neurotransmitters, and the formation of toxic radicals occur thanks to the result of these neurological disorders. The accretion of these toxic mediators further triggers neurodegeneration and, ultimately, death [4]. The homeostatic equilibrium maintained naturally between reactive oxygen species (ROS) and endogenous antioxidants plays a vital role in continuing healthy tissue life. Elevated ROS production predisposes human health to several disorders such as neurodegenerative and inflammatory disorders [5,6,7]. Activation of microglia is another important hallmark in central nervous system (CNS) inflammation, which plays a principal role in immune response in the brain [8]. Activated microglia adopt several morphological forms, each with distinct physiological potential [9]. The up-regulation of inflammatory processes causes the activation of several pro-inflammatory genes, triggering microglial activation and its translocation to the site of injury [10,11,12]. Several studies reportedly demonstrated the role of hyperactivated microglial in oxidative stress and neurodegeneration [13]. Further, it promotes the secretion of several inflammatory markers such as cyclooxygenase-2 (COX2), tumor necrosis factor (TNF-α), nuclear factor κB (NF-κB), interleukin-1β (IL-1β), interleukin-6 (IL-6), and interleukin-10 (IL-10). Such events ultimately trigger neuronal susceptibility and vulnerability to apoptosis [14].

Consistent studies demonstrated the pivotal role of neuroinflammation in ethanol-induced neurodegeneration via several transduction pathways [15]. The exact underlying molecular mechanisms of ethanol-induced neurodegeneration are generally unknown [16], however, several detrimental attributes of ethanol are linked to neurotoxicity [17,18]. Moreover, ethanol is involved in the activation of toll-like receptors (TLRs) and nucleotide-binding oligomerization domain-like receptors, or NOD-like receptors (NLRs), in glial cells, triggering downstream signaling cascade and activating pro-inflammatory cytokines release [19]. These suggested mechanisms make neuronal cells prone to inflammation, and complicate the prognosis of neuropathological disorders [20,21].

Heterocyclic compounds have inspired many scientists to synthesize innovative molecules and to evaluate their biological properties [22,23]. Among heterocyclic ring containing molecules, benzimidazole is one of the most promising moieties [24]. Benzimidazole is becoming a substantial nucleus in medicinal chemistry research because of its high affinity towards a variety of receptors of interest [25,26]. In the current pharmaceutical market, many clinically useful drugs contain benzimidazole nucleus [27]. A number of benzimidazole nucleus-based agents have been proven to have good anti-inflammatory potential [28,29]. Likewise, many compounds containing benzimidazole moiety have been synthesized and evaluated for the treatment of neurodegenerative diseases [30]. The present study aims to evaluate the effects of benzimidazole acetamide derivatives on neuroinflammation and oxidative stress, which could eventually account for cellular protection. If so, the potential molecular and cellular mechanisms underlying these effects still require further delineation. The results obtained will not only help us to understand the cascading mechanisms leading to cell death, but also provide a clue of potential multiple targeting therapeutics.

## 2. Material and Methods

### 2.1. Chemicals

All the starting materials were purchased from Daejung (South Korea) and Alfa-Aesar (Germany). Digital Gallenkamp (Sanyo) apparatus was used to record the melting points of final compounds and was uncorrected. Proton NMR (^1^H-NMR) and carbon-13 (^13^C-NMR) spectra were checked using Bruker AM-300 in DMSO-d6 at 300 MHz and 75 MHz, respectively, while using TMS as an internal standard. Alpha Bruker FTIR spectrophotometer (ATR eco ZnSe, νmax in cm^−1^) was used to record FTIR spectra. All reactions were monitored by thin-layer chromatography (TLC). 3,3-diaminobenzidine peroxidase, an ABC Elite kit, mouse anti-COX-2, mouse anti-p-NF-κB, and mouse anti-Iba-1 were procured from (Santa Cruz Biotechnology, Dallas, TX, USA). H_2_O_2_, Proteinase K, formaldehyde, mounting media, and PBS tablets were acquired from BDH while secondary antibody goat anti-mouse was purchased from Abcam (Cambridge, UK). Other chemicals were procured from Sigma-Aldrich (St. Louis, MO, USA) like solvents and reagents such as 5,5′-dithiobis (2-nitrobenzoic acid), 1-chloro-2,4-dinitrobenzene, glutathione (GSH), N-(1-naphthyl) ethylenediamine dihydrochloride, and trichloroacetic acid. All chemicals used were of high analytical grade (99% HPLC).

### 2.2. General Procedure for the Synthesis of Benzimidazole Acetamide Derivatives (3a and 3b)

**Figure 1:**
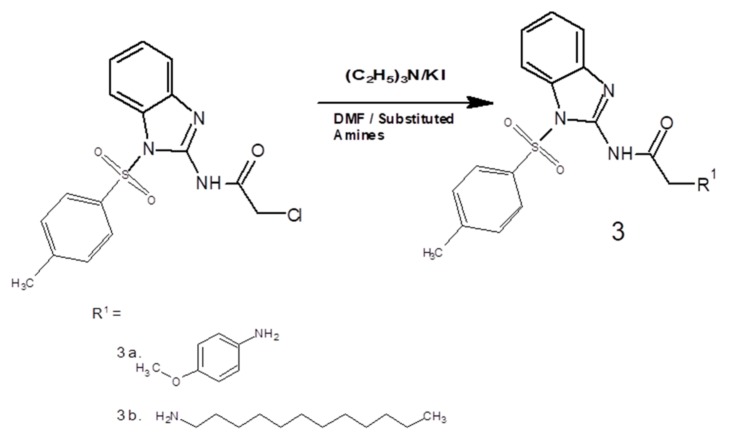
General scheme to synthesize novel benzimidazole acetamide derivatives. DMF, dimethylformamide.

2-amino benzimidazole derivatives 3a and 3b were synthesized following a three-step reaction. Initially, 2-aminobenzimidazole was protected using para toluene sulfonyl chloride (p-TsCl) in the presence of an acetonitrile/water mixture according to a reported method with little modification [31]. In the second step, acetamide was prepared using chloroacetyl chloride (C_2_H_2_Cl_2_O) in anhydrous dichloromethane, CH_2_Cl_2_ (30 mL), at 0 °C. Initially, the reaction mixture was stirred continuously for 15 min on the same temperature, and then it was stirred for further 1 h at ambient temperature/room temperature. Needle-like crystals of crude 2-chloro-N-[1-(4-methylbenzene-1-sulfonyl)-1H-benzimidazol-2-yl] acetamide were obtained, which were further purified through recrystallization in ethanol. The melting point was recorded as 99–102 °C; yield 82% [32]. In the third step, the equimolar ratios of the intermediate product, triethylamine ((CH_3_)_3_N), potassium iodide (KI), and substituted amines (a and b), respectively, were stirred in dimethylformamide (DMF) (30 mL) at room temperature for 5 h (Figure 1: general scheme). The completion of the reaction was assessed by using TLC. The mixture was extracted with ethyl acetate. Anhydrous magnesium sulfate was used to dry the separated organic layer, which was then filtered, and the solvent was removed using a rotary evaporator to obtain the crude final compounds. The purity of the respective final compounds was ensured through silica gel column chromatography [33].

### 2.3. Molecular Docking

The molecular docking studies of synthesized compounds were performed to get an insight into their binding preferences at the active site of the receptors using Autodock Vina software (San Diego, CA, USA, version 4.2.6). The X-ray crystal structures of target proteins COX2 (PDB ID: 5IKQ), TNF-α (PDB ID: 2AZ5), IL-1β (PDB ID: IT4Q), IL-6 (PDB ID: 2I3Y), IL-10 (PDB ID: 2H24), and Iba-1 (PDB ID: 2G2B) were downloaded from http://www.rscb.org/pdb. Active sites of proteins were obtained from DoGSiteScorer, an active site prediction tool [34]. The protein–ligand complexes retrieved from the protein data bank were prepared for docking. Co-crystallized ligands and water molecules were removed from complex and saved as PDB files using Discovery Studio Visualizer. The structures of the ligands were drawn in ChemSketch and saved as Mol files. PDB files of compounds and ligands were generated using Open Babel software [35]. Moreover, proteins and ligands were converted to PDBQT format using AutoDock Tools (version 1.5.6). Furthermore, docking of ligands into active sites of proteins was carried out using Autodock Vina, a digital docking software that is used to interpret results in the form of binding energies [36]. Discovery Studio Visualizer was used to analyze the best binding poses and molecular interactions of ligands in each target protein. The docking procedure was validated by the pose analysis of co-crystallized ligands with the re-docked conformations.

### 2.4. Animals and Drug Treatment

Adult male Sprague Dawley rats weighing between 250 and 290 g (age 10–12 weeks) were acquired from the home-grown facility of Riphah Institute of Pharmaceutical Sciences, Riphah International University (RIU). All the approved protocols were followed as set by the research and ethics committee (REC), RIU. We divided the rats randomly and strictly followed the set criteria to allocate the same weight animals to one group under similar experimental conditions. We divided rats into four groups (12 animal/group) as follows: group I: saline group (control group) (1 mL/kg intraperitoneally for eleven days), group II: ethanol group (2 g/kg intraperitoneally for 11 days), group III: ethanol + compound 3a (ethanol injection (2 g/kg) followed by 3a injection (10 mg /kg) for 11 consecutive days), and group IV: ethanol + compound 3b (ethanol injection (2 g/kg) followed by 3b injection (10 mg/kg) for 11 consecutive days). At the end of the experiment, animals were killed according to the standard protocol (diethyl ether inhalation method). Brain tissues (cortex and hippocampus) were collected and thawed in PBS (pH 7.4 and approximately 5% *w*/*v*). For further analyses, the supernatant solution was separated using a micropipette. To study the histology of tissues, brain tissue after collection were stored in 5% paraformaldehyde, which was later implanted in paraffin, and 4 μm thin coronal sections were made with a rotary microtome (five rats/group).

### 2.5. Behavioral Tests

#### 2.5.1. Y-Maze Test

This test was performed in a special, Y-shaped apparatus. In this maze, each arm measures 50 cm length, 20 cm height, and 10 cm width. Rats were assigned to five groups and each group consisted of six animals. All these animals were injected with a single dose regimen per day for eleven consecutive days. Group I was administered an intraperitoneal dose of saline solution (1 mL/kg) and designated as control. Group II was designated as a disease group that received ethanol (2 g/kg). Animal group III and group IV were assigned as treated groups, in which ethanol + 10 mg/kg dose of drugs 3a and 3b was administered respectively, while Group V was given ethanol + standard drug donepezil (3 mg/kg). A total of three sessions were performed, and each session lasted for 8 min. Briefly, each animal was placed in the middle of the apparatus and permitted to move spontaneously through the maze. A digital video recording camera was used to record all animal entries. The uninterrupted entry of the animals into these three arms was defined as spontaneous alteration behavior. Meanwhile, the percent (%) alteration behavior was defined as follows: successive triplet sets × entries into three different arms successively/total number of arm entries − 2) × 100 [37]. The neurodegenerative potential was assessed by an increase in spontaneous alternation behavior (%).

#### 2.5.2. Morris Water Maze (MWM) Test

In the Morris water maze (MWM) test, all the groups and dosing were kept same as in the Y-maze test. This test was piloted for three continuous days. One the first day, animals were trained for swimming for 60 s in the absence of the probe. For the next two days, a probe was placed in the pool and animals were trained to swim in the presence of this probe. As soon as the animal found that probe, it was allowed to sit on it for 10 s. The rats that failed to locate the probe in 120 s were positioned to stay on the probe for 10 s. After training, the animals were removed from the pool. Finally, on the latter half of day 3, the individual rats were allowed to swim freely in the pool for 120 s in the absence of the probe. Escape latency time was recorded for each animal [37]. The neurodegenerative potential was calculated as a decrease in escape latency time.

### 2.6. Enzyme-Linked Immunosorbent Assays (ELISAs)

p-NF-κB, COX-2, and Iba-1 expression were measured using rat ELISA p-NF-κB (Cat # SU-B28069, Shanghai Yuchun Biotechnology, Shanghai, China), rat COX-2 (Cat # E-EL-M0959, Elabscience Biotechnology Inc., Houston, TX, USA), and rat Iba-1 (Cat # SU-B48003, Shanghai Yuchun Biotechnology, Shanghai, China), respectively, according to the manufacturer’s instructions. Briefly, the supernatants of brain tissue homogenates were collected and protein concentrations were treated with designated antibodies in 96-well plates to quantify p-NF-κB, COX-2, and Iba-1. The concentration of p-NF-κB, COX-2, and Iba-1 were determined using an ELISA microplate reader (BioTekELx808, Biotek, Winooski, VT, USA) after the reaction between the enzyme and substrate. Values were expressed as pictograms of cytokines per milliliter (pg/mL). All the procedures were repeated at least three times.

### 2.7. Morphological Analysis

For morphological analysis, brain samples were first embedded in paraffin blocks and subjected to rotary microtome for trimming and 4 μm thin coronal tissue sections were made, and the following morphological analysis was performed.

#### 2.7.1. Hematoxylin and Eosin (H&E) Staining

Absolute xylene was used for the deparaffinization of the tissue slides and rehydration was done using gradient ethyl alcohol (from 100% to 70%). Distilled water was used further to wash these slides and they were dipped in hematoxylin for 10 min. Subsequently, slides were immersed in water and then washed with 1% ammonia and 1% HCl water. Likewise, the eosin solution was used to treat these slides for 5–10 min. Afterward, these slides were washed with water and dried in air. Dehydration of these slides was done in a graded manner with ethyl alcohol (70%, 95% and 100%), and then they were further washed with xylene, and glass coverslips were placed accordingly. Light microscopy (Olympus, Tokyo, Japan) was used to take the images and ImageJ was used to analyze these images. Three images were recorded per slide. All the images were strained to focus specifically on the inflammatory infiltrated cells, neuron cell size, vacuolation, and neuron cell shapes. The same threshold intensity was adjusted for all the TIF images.

#### 2.7.2. Immunohistochemical Evaluation

Immunohistochemical staining was performed as in the reported method with a few modifications [7]. After deparaffinization and graded hydration, antigens were retrieved by the enzymatic method and then treated with PBS. Then, 3% H_2_O_2_ in methanol was used for blocking endogenous peroxidases for 10 min. Subsequently, incubation of these slides was done with normal goat serum (5%) containing 0.1% Triton X-100, followed by overnight incubation with mouse antibodies (anti-TNFα, anti-Iba-1, and anti-COX2) (dilution 1:100, Santa Cruz Biotechnology, USA). This process continued for the next day. These slides were further incubated in biotinylated secondary antibody (dilution 1:50) followed by washing with 0.1 M PBS solution. The incubation process continued, next with the ABC Elite Kit in a humidified chamber for 1 h. Then, 0.1 M PBS was again used for washing of slides, and staining was done with a solution of 3,3-diaminobenzidine peroxidase. Further, slides were again washed in distilled water and graded ethanol dilutions were finally used for dehydration. These slides were fixed in xylene and coverslips were placed properly using the mounting medium. Light microscopy was used to take the immunohistochemical TIF images.

### 2.8. Oxidative Enzymes Exploration

#### 2.8.1. GSH and GSH S-Transferase (GST) Assay

Reported protocols were used to quantify the level of GSH. Previously diluted and homogenized tissue was added with freshly prepared PBS and then with a solution of 5-5ʹ-dithiobis (2-nitrobenzoic acid). The 412 nm wavelength was used to check the absorbance of this solution. Similarly, the levels of GSH S-transferase (GST) were assayed using reported protocol with little modifications. Concisely, the same concentrations of GST and 1-chloro-2,4-dinitrobenzene were mixed and diluted with a 0.1 M solution of PBS (pH 6.5). After serial dilution from tissue homogenate, the absorbance was measured at 340 nm [6].

#### 2.8.2. Lipid Peroxidation Assay

Lipid peroxide (LPO) assay was performed using a reported method with little modifications [38]. Lipid peroxide level was quantified by measuring the malondialdehyde (MDA) level in the cortical and hippocampal brain tissues homogenate of rats.

#### 2.8.3. Nitric Oxide Assay

Nitric oxide (NO) assay was performed according to the reported Griess reaction with little modifications [39]. Briefly, 50 µl of previously diluted and homogenized tissue and 50 µl of normal saline with an equal volume of Griess reagent (1% sulfanilamide in 0.1% naphthyl ethylenediamine dihydrochloride and 5% phosphoric acid in distilled water) were mixed. The resultant mixture was incubated for 30 min at 37 °C and the absorbance was determined using a microplate reader at 546 nm, with absorbance co-efficient calibrated using standard sodium nitrite solution.

### 2.9. Statistical Analysis

ImageJ was used to analyze the morphological data. Data are evaluated as mean ± SEM and analyzed using the one-way analysis of variance (ANOVA) method followed by post hoc Bonferroni multiple comparisons using Prism GraphPad-6 (San Diego, CA, USA). *p* < 0.05 was considered statistically significant. Symbol # represents a significant difference relative to the saline group, and * represents a significant difference relative to ethanol.

## 3. Results

### 3.1. Spectral Analysis of (3a) [2-(4-methoxyanilino)-N-[1-(4-methylbenzene-1-sulfonyl)-1H-benzimidazol-2-yl] acetamide]

Yield, 89%; m.p., 168–170 °C; Rf = 0.59 (ethyl acetate: n-hexane 1:5); FTIR ν_max_ cm^−1^: 3355(NH), 2957(sp2 CH), 2888 (sp3 CH), 1660 (CO amide), 1589 (C=C aromatic); ^1^H-NMR: δ 2.32 (s, 3H, CH3), 3.34 (s, 2H, CH2), 3.65(s, 3H, OCH3), 4.09 (s, 1H, NH), 6.79 (d, 2H, Ar H, *J* = 9.0 Hz), 6.96 (d, 2H, Ar H, *J* = 8.9 Hz), 7.31 (d, 2H, Ar H, *J* = 8.1 Hz), 7.52 (d, 2H, Ar H, *J* =8.1 Hz), 7.80 (d, 2H, Ar H, *J* = 8.4 Hz), 7.9 (d, 2H, Ar H, *J* = 5.7 Hz); ^13^C-NMR (DMSO-d6, δ ppm); 22.1 (1C, sp3 C), 44.9 (1C, CH2), 55.5 (1C, OCH3), 114.4–115 (4C, Ar), 117.5–125.5 (4C, Ar), 127–129.5 (4C, Ar), 135.5–142.3 (2C, Ar), 140.5 (2C, Ar), 148.5–156.7 (2C, Ar), 154.7 (1C, sp3 C), 169.3 (1C, sp2 C).

### 3.2. Spectral Analysis of (3b) [2-(Dodecylamino)-N-[1-(4-methylbenzene-1-sulfonyl)-1H-benzimidazol-2-yl] acetamide]

Yield, 83%; dark brown viscous liquid; Rf = 0.59 (ethyl acetate: n-hexane 1:5); FTIR ν_max_ cm^−1^: 3355(NH), 2959 (sp2 CH), 2890 (sp3 CH), 1665 (CO amide), 1580 (C=C aromatic); ^1^H-NMR: δ 0.86 (s, 3H, CH3), 1.21–1.35 (m, 20H, 10*CH2, *J* = 7.0 Hz), 1.50 (t, 2H, CH2) 3.31 (s, 2H, CH2), 4.20 (s, 1H, NH), 7.80 (d, 2H, Ar H, *J* = 12.3 Hz), 7.98 (d, 2H, Ar H, *J* = 7.0 Hz), 7.29–7.41(m, 4H, Aromatic); ^13^C-NMR (DMSO-d6, δ ppm); 14 (1C, CH2-N), 21.2 (1C, sp3 C). 22.6–30 (10C, CH2-N), 49.3 (1C, sp3 C), 57 (1C, CO-CH2), 117–126 (4C, Ar), 127–144 (6C, Ar), 140.5 (2C, Ar), 152.7 (1C, sp2 C), 168 (1C, sp2 C).

### 3.3. Docking Evaluation

Synthesized compounds (3a and 3b) along with Co-crystallized ligands were docked into the active sites of COX2, TNF-α, IL1-β, and Iba-1, and the results of docking study are summarized in Table 1.

**Figure 2:**
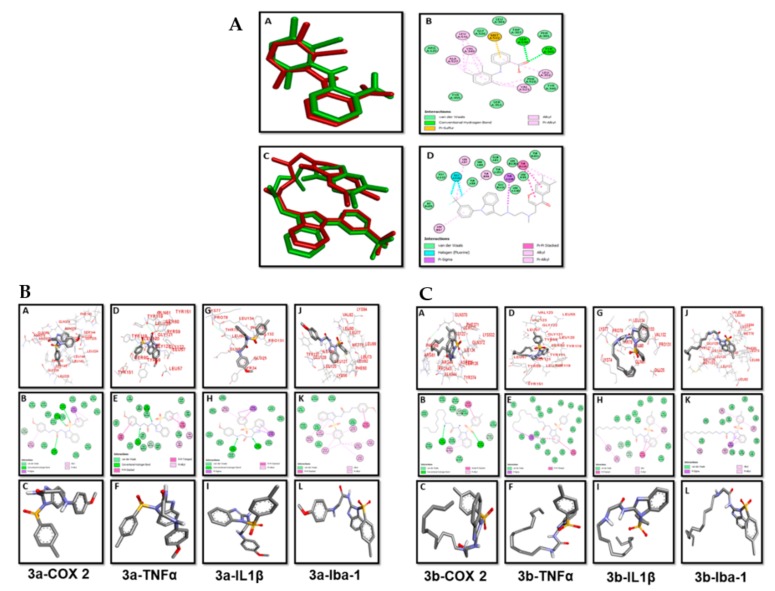
(**A**) Pose analysis and 2D depictions for meclofenamic acid (**A**,**B**) and 6,7-dimethyl-3-[(methyl{2-[methyl({1-[3-(trifluoromethyl)phenyl]-1h-indol-3-yl} methyl)amino]ethyl} amino)methyl]-4h-chromen-4-one (**C**,**D**) in the protein structures of cyclooxygenase-2 (COX-2) and tumor necrotic factor (TNFα), respectively (green = co-crystallized ligand, red = re-docked ligand). (**B**) Post docking analysis visualized by Discovery Studio Visualizer in both 2D and 3D poses. Interactions between 3a and COX2 (**A**,**B**), TNF-α (**D**,**E**), interleukin (IL)-1β (**G**,**H**), and ionized calcium-binding adapter molecule (Iba-1) (**J**,**K**). 3D poses (**A**,**D**,**G**,**J**) and 2D (**B**,**E**,**H**,**K**). (**C**,**F**,**I**,**L**) represents the best pose of 3a that fitted to COX2, TNF-α, IL-1β, and Iba-1, respectively. (**C**) Post docking analysis visualized by Discovery Studio Visualizer in both 2D and 3D styles. Interactions between 3b and COX2 (**A**,**B**), TNF-α (**D**,**E**), IL-1β (**G**,**H**), Iba-1 (**J**,**K**). 3D style (**A**,**D**,**G**,**J**) and 2D style (**B**,**E**,**H**,**K**). (**C**,**F**,**I**,**L**) represents the best pose of 3b that fitted to COX2, TNF-α, IL-1β, and Iba-1, respectively.

Re-docking of the co-crystallized molecules in the same protein was performed for the confirmation and validation of the docking procedure. Figure 2A (A–D) show the poses and binding interactions obtained for the re-docked and co-crystallized ligands in the protein structures of COX2 and TNF-α.

Pose analysis confirmed a near-perfect alignment with the original ligand and displayed the same binding interactions as those obtained from the X-ray crystallographic PDB file. The binding mode and interactions of compound 3a with COX2 are represented in Figure 2B (A–C). Compound 3a contains a para toluene sulfonyl moiety in which the sulfonyl group was seen to form two hydrogen bonds with Gln A:374, Arg A:376 residues, while para methyl and phenyl groups made hydrophobic contacts with Leu145. The oxygen of the amide group was involved in hydrogen bonding with Asn B:376. The attached para-methoxy phenyl substituent was stabilized by hydrophobic interaction with Leu A:146. Figure 2B (D–F) depicted the binding pose of compound 3a with TNF-α. The para toluene sulfonyl group was stabilized with pi–pi stacked interactions with Tyr A:119 and pi–alkyl contacts with Tyr A:151 and Tyr A:59. Two hydrogen bonds were molded, one in between NH (amide) and Leu B:120, and another between the amino group and Gly A:121. Hydrophobic interaction was observed between the phenyl group of para-methoxy phenyl moiety and Tyr B:59.

The lowest energy binding poses and molecular interactions of compound 3a with IL1-β are shown in Figure 2B (G–I). The NH group of amide, as well as amine, was stabilized through hydrogen bonding with amino acid Val A:132 and Leu A:80, respectively. The benzimidazole scaffold and para toluene sulfonyl moiety interacted with Thr A:79 and Phe A:123 by pi–sigma and pi–pi interactions. Ligand–protein interactions of compound 3a with Iba-1 are illustrated in Figure 2B (J–L). Hydrogen bonding was not observed in this case. Benzimidazole and phenyl ring fitted into the active site through hydrophobic interactions with Tyr A:127, Leu A:73, Leu A:77, Ile A:124, and Met A:76. The best binding pose and interactions of compound 3b with COX2 are represented in Figure 2C (A–C). The molecular interactions of compound 3b with COX2 showed two hydrogen bonds, one between the sulfonyl group and Gln A:372, and the second between the NH group and Ile A:124. Moreover, the complex is stabilized by van der Waals force and hydrophobic interactions. Protein–ligand interactions of compound 3b with TNF-α, IL-1β, and Iba-1 highlighted that only hydrophobic interactions were observed in the stabilization of ligand in these targets (Figure 2C (D–L)).

### 3.4. Effect of Compound 3a and 3b on Alternation Behavior

**Figure 3:**
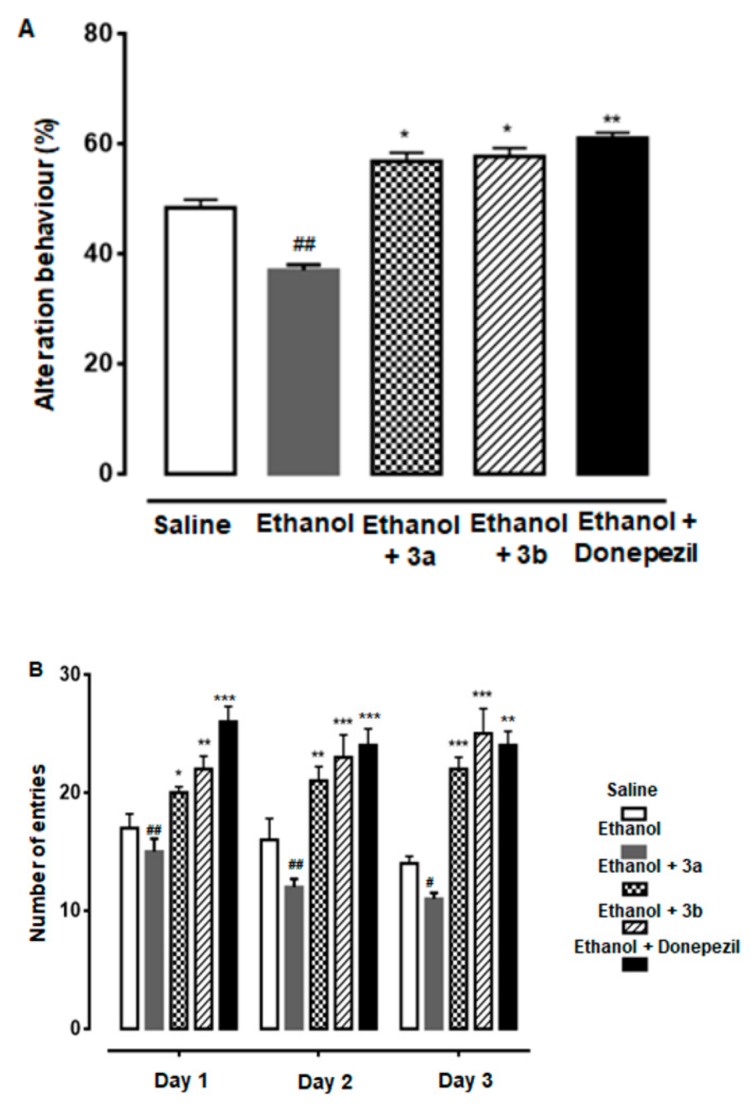
(**A**) Spontaneous alteration behavior % of the rats during the Y-maze test. Mean ± SEM for the rats (*n* = 6/group). ## shows significantly different from the control; *, ** shows significantly different from the ethanol-treated group. Significance: *p* < 0.05. (**B**) Average escape latency time for experimental rats to reach the hidden platform from one to three days. Mean ± SEM for the rats (*n* = 6/group). #, ## shows significantly different from the control; *, **, *** shows significantly different from the ethanol-treated group. Significance: *p* < 0.05.

Both compounds 3a and 3b showed a positive increase in percent spontaneous alternation behavior of rats through an injected dose of 10 mg/kg. In the final trial, the resultant behavior (%) was noted in saline (1 ml/kg), ethanol (2 g/kg), ethanol + 3a (10 mg/kg), ethanol + 3b (10 mg/kg), and ethanol + donepezil (3 mg/kg) treated groups as 47.0 ± 0.6%, 37 ± 0.65%, 57.40 ± 1.0%, 59 ± 2.1%, and 61.0 ± 1.2%, respectively (Figure 3A). All group results were potentially significant (*p* < 0.05 vs. ethanol group).

### 3.5. Effect of Compound 3a and 3b on Escape Latency

The neuroprotective potential was studied by calculating the escape latency time for compounds 3a and 3b in the MWM test in three trials. Both derivatives produced significant results as compared with the ethanol group. Escape latency time observed on day 1 in saline, ethanol, ethanol + 3a, ethanol + 3b, and ethanol + donepezil treated groups were 17.0 ± 1.2, 15 ± 1.1, 20.0 ± 0.5, 22 ± 0.5, and 26 ± 1.3, respectively (Figure 3B). On day 2, it was noted as 16.0 ± 1.8, 12 ± 0.7, 21.0 ± 1.2, 23 ± 1.9, and 24 ± 1.4, respectively, while in the final trial, it was noted as 14.0 ± 0.6, 11 ± 0.5, 22.0 ± 1.0, 25 ± 2.1, and 24 ± 1.2, respectively. All group results were potentially significant (*p* < 0.05 vs. ethanol group).

### 3.6. Effect on Ethanol-Induced Neurodegeneration

**Figure 4:**
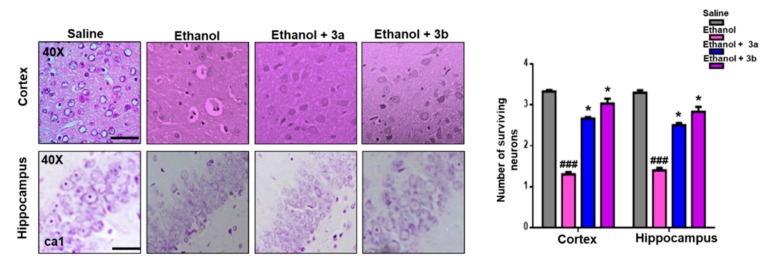
Representative immunohistochemical images of hematoxylin and eosin (H&E) and the quantified histogram of the survival neuron reactivity and integrated density in the cortex and hippocampus region of the adult cortex. The expressed data are relative to the control. ### shows significantly different from the control; ∗ shows significantly different from the ethanol-treated group. Significance: *p* < 0.05. Bar 20 μm, magnification 40× (*n* = 6/group).

Neuroprotective potential of benzimidazole acetamide derivatives 3a and 3b was further demonstrated by H&E staining. The ethanol group showed vigorous morphological changes in the cortex and hippocampus of the rat brain, relative to the saline group (Figure 4, *p* < 0.01). As shown, ethanol produced abnormal morphological features, such as scalloped neuronal shape associated with cytoplasmic eosinophilia/pyknosis and basophilic nature of nuclei. Treatment with compounds 3a and 3b mitigated the ethanol-induced neurotoxicity, and thus more intact cells were noticed in the treated groups (*p* < 0.05, Figure 4).

### 3.7. Pre-Treatment Dosage Regimen Downregulated Neuroinflammation

**Figure 5:**
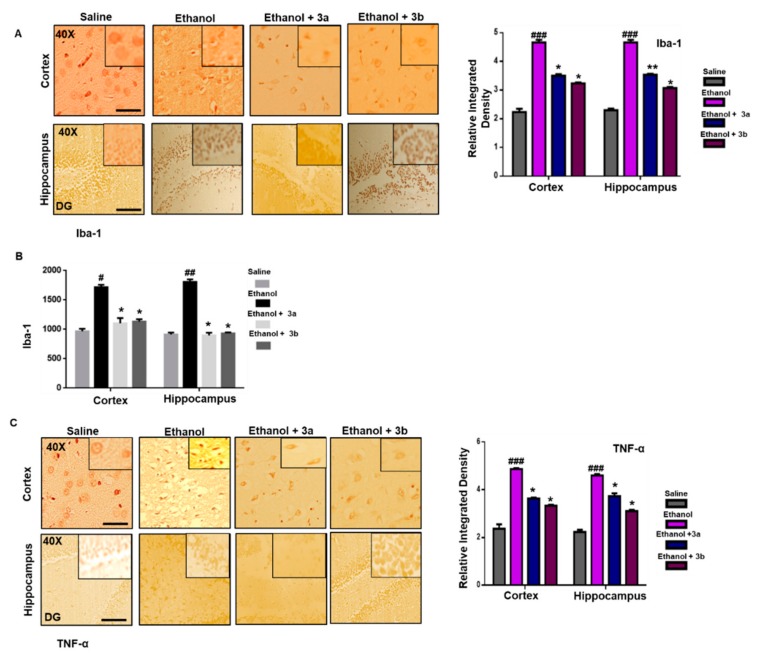
(**A**) Immunohistochemistry results for Iba-1 in the cortex and Dg region of the hippocampus. Bar 20 μm, magnification 40× (*n* = 6/group). Histograms show a comparatively higher expression of Iba-1 in the ethanol group. ** and * show *p* < 0.01 and *p* < 0.05, respectively, and indicate significant difference relative to ethanol, while ^###^ *p* < 0.001 shows significant difference relative to saline group. Data are presented as mean ± SEM. Data are analyzed by one-way analysis of variance (ANOVA) followed by post hoc Bonferroni multiple comparison test using GraphPad Prism 6 software. (**B**) The protein expression of Iba-1 quantified using enzyme-linked immunosorbent assays (ELISA). The data were expressed as the mean ± SEM. * *p* < 0.05 relative to ethanol, while ^#^ *p* < 0.05 and ^##^ *p* < 0.01 relative to saline (*n* = 6/group). (**C**) Immunohistochemistry results of TNF-α in the cortex and Dg region of the hippocampus. Bar 20 μm, magnification 40× (*n* = 6/group). Histograms show a comparatively higher expression of TNF-α in the ethanol group. * shows *p* < 0.05 and indicates significant difference relative to ethanol, while ^###^ *p* < 0.001 shows significant difference relative to the saline group. Data are presented as mean ± SEM. Data are analyzed by one-way ANOVA followed by post hoc Bonferroni multiple comparison test using GraphPad Prism 6 software.

Resident microglia and astrocyte cells critically regulate the inflammation process, as these cells are involved in mediating both detrimental effects by releasing cytokines and protective effects due to brain-derived neurotrophic factor (BDNF) release [40]. The classical sign of microglia activation (Iba-1 reactive cells) is its characteristic hypertrophic appearance, which we revealed by immunohistochemistry. A significant increase in expression of Iba-1 reactive cells was noticed in the ethanol-administered cortex and hippocampus relative to the saline cortex and hippocampus (Figure 5A, *p* < 0.001), which was further validated by ELISA protein assay (Figure 5B, *p* < 0.05). These effects were significantly reversed by pre-treatment with benzimidazole acetamide derivatives (3a and 3b) dosage regimen (Figure 5, *p* < 0.05).

The expression levels of TNF-α (an inflammatory cytokine) were also increased in the ethanol brain, which supports glial cell involvement [41]. Both effects were reversed by pre-treatment with benzimidazole acetamide derivatives (3a and 3b) dosage regimen, as validated by immunohistochemistry findings for TNF-α (Figure 5C, *p* < 0.05).

### 3.8. Pre-Treatment Dosage Regimen Attenuated the Ethanol-Induced Inflammatory Mediators

**Figure 6:**
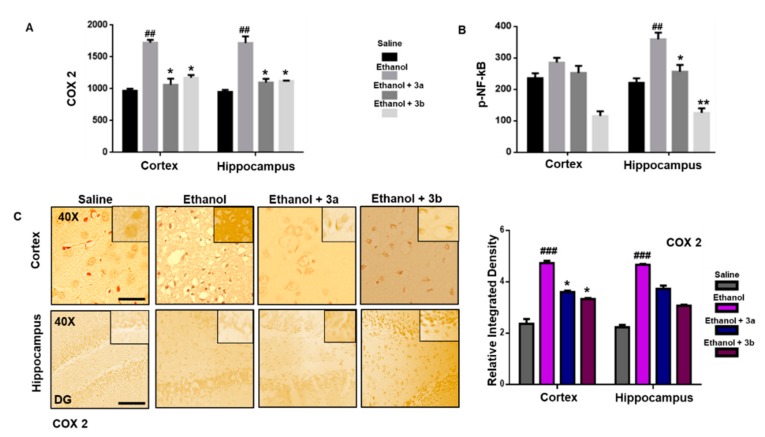
(**A**,**B**) The protein expression of COX2 and p-NF-κB were quantified by ELISA. The data are expressed as the mean ± SEM. * and ** show *p* < 0.05 and *p* < 0.01, respectively, relative to ethanol, while ^##^ *p* < 0.01 relative to saline (*n* = 6/group). (**C**) Immunohistochemistry results of COX2 in the cortex and Dg region of the hippocampus. Bar 20 μm, magnification 40× (*n* = 6/group). COX2 exhibited cytoplasmic localization in both tissues. Histograms show a comparatively higher expression of COX2 in the ethanol group. * shows *p* < 0.05 and indicates significant difference relative to ethanol, while ^###^ *p* < 0.001 shows significant difference relative to saline group. Data are presented as mean ± SEM. Data are analyzed by one-way ANOVA followed by post hoc Bonferroni multiple comparison test using GraphPad Prism 6 software.

The binding of pro-inflammatory factors TNF-α to respective receptors triggers sequential activation of downstream molecules such as apoptosis signal-regulating kinase 1 (ASK1), stress-activated protein/extracellular signal-regulated kinase 1 (SEK1), and c-Jun N-terminal kinase (JNK). Collectively, this leads to proteasomal dependent IκB dissociation, and nuclear translocation of NF-κB to induce inflammatory transcription machinery like COX2 [3]. These proteins were hyper expressed in ELISA results in the ethanol brain (Figure 6A,B, *p* < 0.01). Benzimidazole acetamide derivatives (3a and 3b) pre-treatment dosage regimen significantly alleviated this level (*p* < 0.05). Immunostaining was performed to validate these findings. Similarly, the expression of COX2 was noticed as being significantly high in ethanol-administered brain tissues, while treatment with 3a and 3b reduced this level (Figure 6C, *p* < 0.05).

### 3.9. Effect on Oxidative Enzyme Variations

Table 2 reveals the variation of antioxidant enzymes following ethanol, compound 3a, and compound 3b treatment. Ethanol accumulated the ROS generation in addition to NO (85 ± 6 µg/mL) and alleviated antioxidant enzymes levels such as GSH (3.30 ± 0.97) and GST (2.78 ± 0.45) in the brain cortical tissue (*p* < 0.001). Treatment with 3a and 3b at a dose of 10 mg/kg attenuated downregulation of GSH (34.3 ± 2.4) (40.7 ± 4.1) and GST (28.05 ± 4.3) (35.8 ± 5.1), along with a decreased level of NO (63.15 ± 3.8) (65.2 ± 4.56), respectively, when compared with the ethanol group.

### 3.10. Effect on Ethanol-Induced Lipid Peroxidation (LPO)

Several reports declared malondialdehyde (MDA) as an important indicator to quantify oxidative stress. Thiobarbituric acid reactive substances (TBARS) is a widely used method to assess lipid peroxidation end product malondialdehyde (MDA). This test was performed accordingly, and the results showed a valuable increase in peroxidases after ethanol administration, which could be treated by compounds 3a and 3b. The increased LPO content in the ethanol-treated group was recorded as (224.54 ± 4.32) in comparison with the saline group (*p* < 0.001, Table 2). Benzimidazole acetamide derivatives (3a and 3b) at a dose of 10 mg/kg significantly (Table 2) reduced this content to (160.45 ± 8.9, *p* < 0.05) and (130.8 ± 2.82, *p* < 0.01), respectively.

## 4. Discussion

During the last two decades, a variety of inflammatory mediators have been identified in neurodegenerative brains [42], which might play a pathogenic role in the cortex and hippocampus [43]. The cortex and hippocampus, being more susceptible parts of the brain, are more affected during the early stages of neurodegenerative diseases [44]. One of the important and successful strategies to reduce microglial activation is the use of anti-inflammatory agents, such as drugs that inhibit COX2, which reduces the neuroinflammation and improves behavioral deficits [45]. Furthermore, this strategy protects against neurodegeneration in the hippocampus of an ethanol-exposed rat brain [46]. As the benzimidazole nucleus has a strong affinity towards multiple targets, toxicological studies have proven it as a safer moiety for future drug design and development [47]. In the last few decades, the benzimidazole nucleus has been reported in many pharmaceutical patents owing to its high pharmacological value [48]. Similarly, many compounds containing benzimidazole nucleus have been reported as potential anti-inflammatory agents [49,50]. Therefore, in our study, we established the synthesis of two novel anti-inflammatory substituted benzimidazole compounds, which effectively improved the neuroprotective effects through boosted antioxidant effect, as well as reduced oxidative stress and neuroinflammation. Many established reports demonstrated that the benzimidazole nucleus-containing drugs are highly suitable to produce beneficial effects against neuronal death in many neurodegenerative disease models [51,52,53]. Reportedly, benzimidazole derivatives act not only as an endogenous potent anti-oxidant and free radical scavenger in various pathological and disease conditions [54,55], but also as an activator of many antioxidant systems. In particular, they activate superoxide dismutase (SODs), GSH, Sirtuins (SIRTs), and nuclear factor erythroid 2-related factor 2/heme oxygenase-1 (Nrf2/HO-1) pathway. Many published reports evidently show a relationship between the activation of inflammatory cascades and the accretion of oxidative stress [56,57].

These new compounds were synthesized by using the general scheme, shown in Figure 1, with little modifications. The 2-aminobenzimidazole was converted to 1-[dihydroxy(4-methyl phenyl)-λ4-sulfanyl]-1H-benzimidazol-2-amine by using para toluene sulfonyl chloride in the presence of acetonitrile and water. Further, it was reacted with CH_2_Cl_2_ in the presence of triethylamine and dried solvent (methylene chloride) to convert it into 2-chloro-N-[1-(4-methylbenzene-1-sulfonyl)-1H-benzimidazol-2-yl] acetamide. FTIR results confirmed the formation of intermediate compounds through the generation of new bonds such as amide bond stretch at 1660 cm^−1^ (–CO) and stretching of (–NH) at 3355 cm^−1^. The resultant derivatives (3a and 3b) were prepared by the nucleophilic reaction of the intermediate compound and primary amines (p-anasidine and dodecylamine), respectively. One singlet of amide proton at δ = 4.09 confirmed the formation of the amide bond in the intermediate product. Final confirmation of the resultant derivatives was confirmed by FTIR, ^1^H-NMR, and ^13^C-NMR spectral results.

Neuroinflammation is directly linked with ROS provoked oxidative stress, which further aggravates neurodegenerative disease pathogenesis. Neuroinflammation promotes the activation of several inflammatory cytokines and mediators (COX2), inducible nitric oxide synthase (iNOS), IL-6, IL-10, TNF-α, mitogen-activated protein kinases (MAPKs), nuclear factor-κB (NF-κB) activation, and microglial activation (Iba-1) [58,59]. Treatment using our newly synthesized compounds produced a profound neuroprotective effect by strengthening the intrinsic antioxidant effect (GSH, GST) and also by attenuating proinflammatory cytokines and ROS generation. In different reported scenarios, increased ROS production owing to oxidative stress and activated microglia presents an increased risk of many morphological changes and neurodegeneration in the brain. In different repetitive trials, many neuroprotective agents targeting neuroinflammation failed as a result of increased side effects, lesser central nervous system (CNS) permeability, and reduced therapeutic activity because of usually targeting a single site [60]. Our results demonstrated that a neuroprotectant with less hydrogen bond donors and lesser polar surface area has a rapid CNS penetration and can act on multiple targets to attenuate the oxidative stress-induced neuroinflammation. We hypothesize that our newly synthesized derivatives can reduce neuronal injury by modulating cytokines’ expression and by downregulating the p-NF-κB pathway.

## 5. Conclusions

In conclusion, ethanol injury activates several proinflammatory mediators including NF-κB and is further linked to ROS generation. Our newly synthesized compounds attenuated ethanol-induced oxidative stress and inflammatory cascade, possibly by modulating the ROS/TNF-α/NF-κB/COX2 pathway, eventually accounting for its neuroprotective effects against neuronal apoptosis.

## Figures and Tables

**Table 1:** Binding energy values after docking. IL, interleukin; TNF, tumor necrotic factor, COX, cyclooxygenase; Iba, ionized calcium-binding adapter molecule.

Compounds	COX2	TNF-α	IL-1β	Iba-1
Binding Energies (kcal/mol)				
3a	–8.9	–8.4	–7.6	–7.3
3b	–7.4	–7.4	–6	–7
Co-crystal	–9.1	–9.3	-	-

**Table 2:** Effect of 3a and 3b on oxidative enzymes.

Group	GSH (mg/1100 g Tissue)	GST (pmol)	i-NOS (pmol)	LPO
Saline	54.88 ± 3.78	40.42 ± 1.30	21.02± 7.61	79.32 ±0.70
Ethanol	3.30 ± 0.97 ^###^	2.78 ± 0.45 ^###^	85 ± 6 ^###^	224.54 ± 4.32 ^###^
Ethanol + 3a	34.3 ± 2.4 **	28.05 ± 4.3 **	63.15 ± 3.8 *	160.45 ± 8.9 *
Ethanol + 3b	40.7 ± 4.1 **	35.8 ± 5.1 ***	65.2 ± 4.56 *	130.8 ± 2.82 **

Symbols *** or ^###^ show significant difference at *p* < 0.001, while * and ** show significant difference at *p* < 0.05 and *p* < 0.01, respectively. All data were analyzed by one-way analysis of variance (ANOVA) followed by post hoc Bonferroni multiple comparison test. Data are presented as mean ± SEM. Symbol * shows a significant difference relative to ethanol and # shows significant difference relative to saline. Abbreviations: GST, glutathione S-transferase; GSH, glutathione; TBARS, thiobarbituric acid reactive substances; i-NOS, inducible nitric oxide synthase; LPO, lipid peroxidation.
